# Prediction model for successful liberation from continuous renal replacement therapy in patients with acute kidney injury: development and temporal validation

**DOI:** 10.3389/fmed.2026.1882567

**Published:** 2026-07-30

**Authors:** Zengyan Hu, Xi Yu, Xiaoche Liu, Tingjiang Jiang, Liang Chen

**Affiliations:** 1 Department of Emergency Medicine, Xinhua Hospital, School of Medicine, Shanghai Jiao Tong University, Shanghai, China; 2 Department of Nephrology, Ruijin Hospital, School of Medicine, Shanghai Jiao Tong University, Shanghai, China

**Keywords:** acute kidney injury, continuous renal replacement therapy, liberation, prediction model, temporal validation

## Abstract

**Background:**

The optimal timing of liberation from continuous renal replacement therapy (CRRT) in adults with acute kidney injury (AKI) remains uncertain. This study developed and temporally validated a model for predicting successful 7-day liberation after a planned CRRT discontinuation attempt and compared its performance with urine output and serum creatinine (SCr)-based indices.

**Methods:**

This single-center retrospective cohort included 447 adults with AKI who underwent a first planned CRRT discontinuation attempt between 2019 and 2024. Successful liberation was defined as survival without kidney replacement therapy (KRT) for 7 consecutive days after CRRT discontinuation. Patients treated in 2019–2022 formed the development cohort (*n* = 304), and those treated in 2023–2024 formed the temporal validation cohort (*n* = 143). Six prespecified predictors—Sequential Organ Failure Assessment (SOFA) score, sepsis-associated AKI, preceding 24-h urine output, relative SCr decline over the preceding 24 h, time-weighted mean arterial pressure (MAP), and vasoactive drug use—were entered into a multivariable logistic regression model. Multiple imputation, bootstrap optimism correction, calibration assessment, Brier score, decision curve analysis, and sensitivity analyses were performed.

**Results:**

Overall, 241 patients (53.91%) achieved successful 7-day liberation. The optimism-corrected area under the receiver operating characteristic curve (AUC) in the development cohort was 0.860 (95% confidence interval [CI], 0.817–0.900), and the temporal validation AUC was 0.853 (95% CI, 0.785–0.913). In temporal validation, the Brier score was 0.156 (95% CI, 0.123–0.192), calibration intercept was −0.225 (95% CI, −0.648 to 0.198), and calibration slope was 0.984 (95% CI, 0.654–1.313). At the fixed development-derived threshold of 0.529, sensitivity was 84.21% and specificity was 71.64%. The full model outperformed urine output alone and SCr decline alone, whereas its AUC did not differ significantly from the combined urine output plus SCr model.

**Conclusion:**

This model provides individualized 7-day liberation risk estimates after standard CRRT discontinuation criteria and checklist completion. It is intended as an adjunctive risk-stratification tool for experienced clinicians and should not replace clinical judgment. Prospective multicenter external validation, local recalibration when needed, and clinical impact evaluation are warranted before implementation.

## 1 Introduction

Acute kidney injury (AKI) is a common organ dysfunction among hospitalized and critically ill patients and is associated with increased mortality, incident chronic kidney disease, and greater healthcare resource use (1). Continuous renal replacement therapy (CRRT) is widely used in critically ill patients who require kidney support, although patient case mix, treatment prescriptions, and outcomes vary substantially across centers (2). CRRT allows continuous and gradual control of solutes, volume, electrolytes, and acid–base balance, particularly in patients with hemodynamic instability (3). No unified standard has been established for the optimal timing of CRRT discontinuation; premature discontinuation may lead to the need for renewed kidney replacement therapy (KRT), whereas unnecessary delay prolongs exposure to vascular catheters, anticoagulation, and resource consumption (4).

Previous studies have evaluated urine output, serum creatinine (SCr) trajectories, creatinine clearance, and diuretic response, but thresholds and measurement windows have varied widely (5). Multivariable models based on routinely available clinical variables have shown predictive ability (6), and existing models have undergone independent validation in multicenter cohorts (7). More recently, machine learning models based on large intensive care databases have expanded the range of candidate predictors (8), although some models remain limited by short outcome windows, incomplete calibration reporting, or dependence on local clinical workflows (9). Interpretable machine learning studies suggest that complex models may improve discrimination, but their incremental value and stability across settings require direct evaluation (10).

The primary outcome of this study was survival without any form of KRT for 7 consecutive days after CRRT discontinuation. We developed the model using each patient’s first planned CRRT discontinuation attempt and performed temporal validation in a subsequent cohort from the same center. We also compared the full model with urine output, SCr, and combined urine output plus SCr models; reported detailed calibration and overall accuracy; and assessed robustness in analyses excluding decision-related variables, using a 72-h outcome, restricting the sample to protocol-adherent or complete cases, and including all repeated attempts. The intended users are nephrology and critical care clinicians with experience in CRRT management. The model is intended for individualized risk stratification after standard CRRT discontinuation criteria have been fulfilled and the discontinuation checklist has been completed, serving as an adjunctive decision-support tool that provides an estimated probability of successful 7-day liberation and should not replace clinical judgment. Actual decisions should integrate volume status, electrolytes, acid–base balance, hemodynamic stability, and the patient’s overall goals of care.

## 2 Materials and methods

### 2.1 Study design and participants

This study was reviewed and approved by the Medical Ethics Committee of Ruijin Hospital, Shanghai Jiao Tong University School of Medicine (approval number: NO. KY2026-1020). The ethics committee approved the use of de-identified retrospective medical record data and waived the requirement for individual written informed consent. No prospective intervention, additional biological sample collection, or change in clinical care pathways was performed. All study procedures complied with institutional ethical requirements and the Declaration of Helsinki.

We retrospectively screened adult patients with AKI who received CRRT between January 2019 and December 2024. AKI diagnosis and staging followed the Kidney Disease: Improving Global Outcomes (KDIGO) criteria (11); sepsis-associated AKI was defined according to Sepsis-3 (12); and chronic kidney disease (CKD) stages 1–4 were determined from estimated glomerular filtration rate (eGFR), proteinuria, or structural kidney abnormalities documented at least 3 months before admission (13).

The inclusion criteria were age ≥18 years, CRRT duration ≥24 h, at least one planned CRRT discontinuation attempt during hospitalization, and ascertainable primary outcome status. The exclusion criteria were age <18 years, CKD stage 5 or maintenance dialysis, kidney transplantation, CRRT duration <24 h, CRRT initiated at another institution before admission, no planned discontinuation attempt before death or transfer, and nonmedical treatment withdrawal with an indeterminate 7-day outcome. Exclusion reasons were counted in a mutually exclusive sequence in the order listed.

The primary unit of analysis was the patient, and only the first planned CRRT discontinuation attempt during hospitalization was included for each patient. All repeated attempts were included in a sensitivity analysis using patient-clustered generalized estimating equations (GEE). According to the actual CRRT discontinuation date, patients treated in 2019–2022 were assigned to the development cohort (*n* = 304), and those treated in 2023–2024 were assigned to the temporal validation cohort (*n* = 143).

Reporting followed the Transparent Reporting of a multivariable prediction model for Individual Prognosis Or Diagnosis (TRIPOD) statement (14). The Prediction model Risk Of Bias ASsessment Tool (PROBAST) (15) was used to evaluate risk of bias and applicability for model development and temporal validation.

### 2.2 CRRT protocol, discontinuation checklist, and KRT restart

Before CRRT discontinuation, the Sequential Organ Failure Assessment (SOFA) score was used to characterize multiple-organ dysfunction (16). CRRT was delivered through a double-lumen catheter inserted in the femoral or internal jugular vein. Treatment modes included continuous venovenous hemofiltration (CVVH), continuous venovenous hemodialysis (CVVHD), and continuous venovenous hemodiafiltration (CVVHDF). The prescribed blood flow rate was usually 150–200 mL/min, and the delivered dialysate/replacement fluid flow rate was 25–35 mL/(kg·h). Indications for KRT initiation included refractory hyperkalemia, severe metabolic acidosis, uremic complications, refractory fluid overload, and persistent oliguria or anuria with inability to maintain volume, electrolyte, or acid–base homeostasis (17).

The following checklist items were assessed before planned CRRT discontinuation: urine output ≥0.5 mL/(kg·h) for at least 12 h; stable or decreasing SCr during the preceding 24 h; volume status maintainable without KRT and no progressive pulmonary edema; serum potassium <5.5 mmol/L, pH > 7.25, and no progressive uremic manifestations; mean arterial pressure (MAP) ≥ 65 mmHg, with a stable or decreasing vasoactive drug dose during the preceding 12 h; and confirmation by the attending physician. Protocol adherence was defined as all items being fulfilled and documented before CRRT discontinuation. Any clearly unmet item was classified as protocol deviation, and any key item that could not be verified was classified as insufficient documentation. The time when all criteria were first fulfilled was recorded as tcriteria, the time of actual CRRT discontinuation was recorded as t0, and decision delay was defined as t0 − tcriteria. Conservatively, cases with insufficient documentation were counted as unconfirmed adherence.

KRT restart was based on the same physiological indications as initial KRT initiation, but only when abnormalities after discontinuation could not be corrected by conventional medical or volume management. Any form of KRT could be restarted for refractory hyperkalemia (serum potassium ≥6.0 mmol/L, or a rapid increase with electrocardiographic changes); severe metabolic acidosis (pH still ≤7.20 after conventional treatment); refractory fluid overload or pulmonary edema causing hypoxemia; uremic complications such as pericarditis or encephalopathy; or persistent oliguria/anuria with progressive azotemia and inability to maintain volume, electrolyte, or acid–base homeostasis. KRT restart and modality selection were jointly determined by the attending nephrologist and intensive care unit (ICU) physician. An increase in SCr or decrease in urine output triggered clinical reassessment. Restart criteria were consistent with the physiological principles emphasized in clinical guidelines for severe AKI (18).

Regional citrate anticoagulation, heparin anticoagulation, or no anticoagulation was selected according to bleeding risk (19).

### 2.3 Data collection, time windows, and outcomes

The index time, t0, was defined as the actual time of CRRT discontinuation. All predictors were obtained before t0. Cumulative urine output, fluid intake, and fluid output were measured within the [t0–24 h, t0) window; MAP was calculated as the time-weighted mean of electronic monitoring records during the same window; vasoactive drug use was defined as any continuous infusion during this window; and SOFA score was calculated from the worst values in the six organ systems during [t0–24 h, t0), using the highest organ-specific score when multiple measurements were available. Relative SCr decline during the last 24 h was calculated as {[SCr(t0–24 ± 4 h) − the last measured SCr within 0–4 h before t0]/SCr(t0–24 ± 4 h)} × 100%. Data obtained after t0 were used only for outcome ascertainment.

Baseline variables included age, sex, body mass index (BMI), comorbidities, Acute Physiology and Chronic Health Evaluation II (APACHE II) score, SOFA score, AKI stage and etiology, baseline SCr, and urine output before CRRT initiation. Treatment variables included CRRT mode, anticoagulation strategy, duration, blood flow rate, delivered dialysate/replacement fluid flow rate, net ultrafiltration volume, and filter replacement count. Variables during the 24 h before CRRT discontinuation included urine output, fluid balance, SCr change, MAP, vasoactive drug use, mechanical ventilation, and the ratio of arterial partial pressure of oxygen to fraction of inspired oxygen (PaO₂/FiO₂). Dynamic pre-discontinuation measurements of cystatin C, neutrophil gelatinase-associated lipocalin (NGAL), tissue inhibitor of metalloproteinases-2 (TIMP-2), and insulin-like growth factor-binding protein 7 (IGFBP7) were not collected under a standardized protocol.

The primary outcome was successful 7-day liberation from CRRT, defined as survival without any form of KRT for 7 consecutive days from the actual CRRT discontinuation time (t0). KRT included repeat CRRT, intermittent hemodialysis, prolonged intermittent kidney replacement therapy, and peritoneal dialysis. Liberation failure was determined by actual restart of any KRT or all-cause death within 7 days. Post-discontinuation SCr elevation or reduced urine output triggered clinical reassessment but did not itself define failure. The original 72-h outcome was retained as a prespecified sensitivity analysis. The definition of sustained 7-day liberation was consistent with recent consensus on CRRT liberation (20).

Secondary outcomes were 28-day all-cause mortality, renal recovery among 28-day survivors, and chronic dialysis dependence among 90-day survivors. Complete recovery was defined as SCr ≤ 1.5 times the baseline value. Partial recovery was defined as not meeting the criterion for complete recovery and having an SCr decrease of ≥50% from CRRT initiation. No recovery was defined as an SCr decrease of <50%. Vital status and KRT status at 28 and 90 days were complete.

Electronic medical record data were extracted according to a prespecified data dictionary and standardized to common units. Range, logic, and temporal sequence checks were performed, and outliers were verified against source records. Sepsis-associated AKI status, CKD status, and SOFA score were independently reviewed by two trained investigators according to prespecified criteria and blinded to the 7-day outcome; other predictors were obtained from electronic information systems or derived according to prespecified calculation rules. The primary outcome was independently adjudicated by two investigators using KRT orders, treatment records, and death records. Disagreements were resolved by a third nephrologist. Model-predicted probabilities were not used for outcome adjudication.

### 2.4 Statistical analysis

Statistical analyses were performed using R version 4.3.0 and SPSS version 26.0. The normality of continuous variables was assessed using the Shapiro–Wilk test and distribution plots. Approximately normally distributed variables are presented as mean ± standard deviation and were compared using the Welch two-sample *t* test; nonnormally distributed variables are presented as median (interquartile range) and were compared using the Mann–Whitney *U* test. Categorical variables are presented as counts (percentages) and were compared using the Pearson *χ*^2^ test; Fisher’s exact test was used when any expected cell count was <5. The sample size was determined by all eligible patients during the study period. The smaller outcome category in the development cohort included 139 patients, corresponding to approximately 23.2 observations per parameter for six model parameters. The development and temporal validation cohorts were first separated by CRRT discontinuation date, after which 20 datasets were independently generated within each cohort using multiple imputation by chained equations. The imputation model included the outcome, the six predictors, and auxiliary variables related to missingness; predictive mean matching was used for continuous variables, and the corresponding logistic models were used for categorical variables. The model was fitted in each imputed development dataset, and coefficients and variances were pooled using Rubin’s rules. The six predictors were fixed before refitting and entered simultaneously into the multivariable logistic regression model. Restricted cubic splines were used to examine functional form; when no clinically meaningful nonlinearity was detected, continuous variables were modeled linearly. In each imputed development dataset, 1,000 bootstrap resamples were used to estimate optimism and a uniform shrinkage factor, after which the intercept was re-estimated. The final locked equation was applied directly to the temporal validation cohort without variable reselection, coefficient updating, or threshold optimization. Model performance measures included area under the receiver operating characteristic curve (AUC), Brier score, calibration curve, calibration intercept, calibration slope, observed-to-expected ratio (O: E), and 95% confidence interval (CI). The threshold maximizing the Youden index in the development cohort was fixed and used to calculate sensitivity, specificity, positive predictive value, and negative predictive value in the validation cohort. Confidence intervals for validation performance were estimated using 2,000 patient-level bootstrap resamples, with imputation and estimation repeated within each resample. Decision curve analysis (DCA) assessed net benefit over threshold probabilities of 0.25–0.75. Prespecified analyses included simple marker models, a model excluding workflow-related variables, the 72-h outcome, an outcome restricted to KRT restart, protocol-adherent cases, complete cases, absolute SCr change, fixed median/mode imputation, patient-clustered GEE for all 603 attempts, and prespecified subgroups. All tests were two-sided, and *p* < 0.05 was considered statistically significant. Variable-level missingness, cohort comparison, repeated-attempt analysis, subgroup analysis, and reporting-standard assessment are provided in Supplementary Tables S1–S6.

## 3 Results

### 3.1 Patient flow, primary outcome, and baseline characteristics

**Figure 1:**
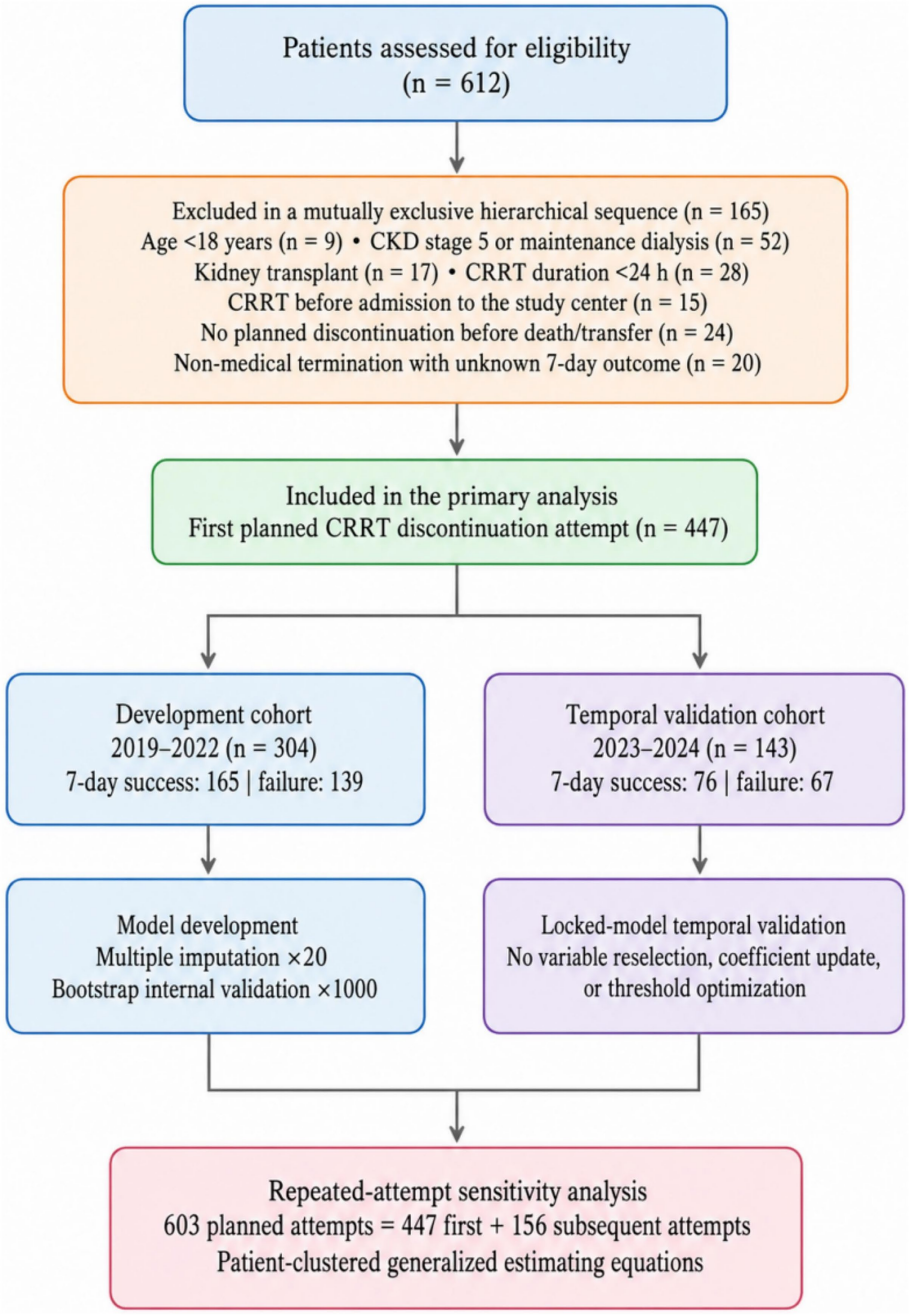
Patient screening, cohort allocation, and analysis flow. Exclusion reasons were counted in a mutually exclusive prespecified hierarchy. The primary analysis included the first planned continuous renal replacement therapy (CRRT) discontinuation attempt in 447 patients; 156 subsequent attempts were included in the sensitivity analysis using patient-clustered generalized estimating equations (GEE). *n*, sample size; CKD, chronic kidney disease.

A total of 612 patients were screened, of whom 165 were excluded in a mutually exclusive hierarchical sequence: age <18 years (*n* = 9), CKD stage 5 or maintenance dialysis (*n* = 52), kidney transplantation (*n* = 17), CRRT duration <24 h (*n* = 28), CRRT initiated at another institution before admission (*n* = 15), no planned discontinuation attempt before death or transfer (*n* = 24), and nonmedical treatment withdrawal with an indeterminate 7-day outcome (*n* = 20). The final analysis included 447 patients, each contributing one first planned CRRT discontinuation attempt. According to the 7-day primary outcome, 241 patients (53.91%) achieved successful liberation and 206 (46.09%) failed; among those with failure, 190 restarted KRT and 16 died before restart. The main indications among the 190 patients who restarted KRT were refractory fluid overload/pulmonary edema (*n* = 72), severe metabolic acidosis (*n* = 41), refractory hyperkalemia (*n* = 38), persistent oliguria/anuria with progressive azotemia (*n* = 31), and uremic complications (*n* = 8). Under the original 72-h definition, 268 patients were classified as successfully liberated, among whom 27 experienced delayed failure on days 4–7. Predictor missingness ranged from 0 to 9.17%, and the primary outcome had no missing data (Figure 1 and Supplementary Table S1).

Age, male sex, BMI, hypertension, diabetes mellitus, chronic heart failure, chronic liver disease, malignancy, and surgery within 30 days did not differ significantly between groups (all *p* > 0.05). The failure group had higher proportions of CKD stages 1–4 and sepsis-associated AKI, higher APACHE II and SOFA scores, a more severe distribution of AKI stages, and higher baseline SCr, whereas the success group had greater urine output at ICU admission and during the 24 h before CRRT initiation (all *p* < 0.05) (Table 1).

**Table 1:** Baseline and key continuous renal replacement therapy (CRRT) initiation characteristics by outcome group.

Variable	Success group (*n* = 241)	Failure group (*n* = 206)	Statistic	*p*-value
Age (years)	61.37 ± 13.87	62.92 ± 14.81	Welch *t* = −1.136	0.257
Male sex	145 (60.17)	131 (63.59)	*χ*^2^ = 0.552	0.458
BMI (kg/m^2^)	24.10 ± 3.79	23.83 ± 3.91	Welch *t* = 0.738	0.461
Hypertension	114 (47.30)	106 (51.46)	*χ*^2^ = 0.767	0.381
Diabetes mellitus	77 (31.95)	80 (38.83)	*χ*^2^ = 2.310	0.129
Chronic heart failure	39 (16.18)	44 (21.36)	*χ*^2^ = 1.968	0.161
Chronic liver disease	27 (11.20)	33 (16.02)	*χ*^2^ = 2.217	0.137
Malignancy	36 (14.94)	40 (19.42)	*χ*^2^ = 1.579	0.209
Surgery within 30 days	91 (37.76)	65 (31.55)	*χ*^2^ = 1.883	0.170
CKD stages 1–4	42 (17.43)	56 (27.18)	*χ*^2^ = 6.177	0.013
APACHE II score	18.98 ± 6.10	23.00 ± 7.00	Welch *t* = −6.419	<0.001
SOFA score	8.67 ± 3.21	11.21 ± 3.37	Welch *t* = −8.118	<0.001
AKI stage: 1/2/3	27/81/133	9/49/148	*χ*^2^ = 15.029	<0.001
Sepsis-associated AKI	91 (37.76)	118 (57.28)	*χ*^2^ = 17.003	<0.001
Baseline SCr (μmol/L)	80.1 (66.8, 95.0)	85.4 (72.0, 103.5)	*Z* = −2.344	0.019
Urine output at ICU admission (mL/h)	26.6 (13.7, 48.6)	18.1 (9.4, 35.2)	*Z* = 4.049	<0.001
Urine output during 24 h before CRRT initiation (mL)	390 (197, 725)	264 (119, 534)	*Z* = 3.745	<0.001

Continuous variables are presented as mean ± standard deviation (SD) or median (interquartile range), as appropriate; categorical variables are presented as counts (percentages) [*n* (%)]. Welch t denotes the Welch two-sample t statistic, *χ*^2^ denotes the Pearson chi-square statistic, *Z* denotes the standardized Mann–Whitney U statistic, and *p* denotes the two-sided *p*-value. BMI, body mass index; CKD, chronic kidney disease; APACHE II, Acute Physiology and Chronic Health Evaluation II; SOFA, Sequential Organ Failure Assessment; AKI, acute kidney injury; SCr, serum creatinine; ICU, intensive care unit; CRRT, continuous renal replacement therapy. All tests were two-sided.

The development and validation samples did not differ significantly in 7-day success rate, age, male sex, CKD stages 1–4, sepsis-associated AKI, SOFA score, cumulative urine output during the 24 h before discontinuation, relative SCr decline during the last 24 h, 24-h time-weighted mean MAP, vasoactive drug use, or CRRT mode distribution (all *p* > 0.05) (Supplementary Table S2).

### 3.2 Protocol implementation and key 24-h measures before CRRT discontinuation

Among the 447 patients, records supported protocol adherence in 404 (90.38%), 28 had explicit protocol deviations (17 for urine output criteria and 11 for hemodynamic criteria), and 15 had insufficient documentation of key criteria; conservatively, cases with insufficient documentation were classified as unconfirmed adherence. The mean interval from first fulfillment of all criteria to actual CRRT discontinuation was 7.3 ± 5.0 h. The success group had a higher protocol adherence rate and a shorter interval from fulfillment of all CRRT discontinuation criteria to actual discontinuation. The success group also had higher mean hourly urine output, 24-h cumulative urine output, total fluid output during the preceding 24 h, relative SCr decline during the last 24 h, 24-h time-weighted mean MAP, and PaO₂/FiO₂, whereas 24-h total intake, positive fluid balance, vasoactive drug use, mechanical ventilation, and CRRT duration were lower (all *p* < 0.05). Delivered dialysate/replacement fluid flow rate did not differ significantly between groups (*p* = 0.228) (Table 2).

**Table 2:** Protocol implementation and key measures during the 24 h before continuous renal replacement therapy (CRRT) discontinuation.

Variable	Success group (*n* = 241)	Failure group (*n* = 206)	Statistic	*p*-value
Protocol adherence	224 (92.95)	180 (87.38)	*χ*^2^ = 3.960	0.047
Time from criteria fulfillment to treatment discontinuation (h)	6.7 ± 4.4	8.1 ± 5.5	Welch *t* = −2.94	0.004
Mean hourly urine output (mL/(kg·h))	0.77 (0.49, 1.25)	0.41 (0.26, 0.63)	*Z* = 8.949	<0.001
24 h cumulative urine output (mL)	1,262 (783, 2027)	606 (433, 1,053)	*Z* = 9.260	<0.001
24 h fluid intake (mL)	3256.1 ± 841.6	3426.1 ± 908.5	Welch *t* = −2.039	0.042
24 h fluid output (mL)	3121.1 ± 1379.1	2575.6 ± 1263.4	Welch *t* = 4.362	<0.001
24 h fluid balance (mL)	135.0 ± 1023.9	850.5 ± 1077.4	Welch *t* = −7.160	<0.001
Relative SCr decline during the last 24 h (%)	24.2 ± 16.5	11.3 ± 16.4	Welch *t* = 8.266	<0.001
24 h time-weighted mean MAP (mmHg)	76.0 ± 11.7	69.8 ± 11.9	Welch *t* = 5.533	<0.001
Vasoactive drug use	124 (51.45)	157 (76.21)	*χ*^2^ = 29.169	<0.001
Mechanical ventilation	125 (51.87)	162 (78.64)	*χ*^2^ = 34.642	<0.001
PaO₂/FiO₂ (mmHg)	245.0 ± 68.0	188.6 ± 71.7	Welch *t* = 8.490	<0.001
CRRT duration (h)	75 (43, 114)	101 (69, 157.5)	*Z* = −5.582	<0.001
Delivered dialysate/replacement fluid flow rate (mL/(kg·h))	28.7 ± 4.2	29.2 ± 4.5	Welch *t* = −1.207	0.228

t0 denotes the actual discontinuation time of continuous renal replacement therapy (CRRT). Cumulative and mean 24-h variables were calculated during [t0–24 h, t0), and relative serum creatinine decline during the last 24 h was calculated using the t0–24 ± 4 h measurement and the last measurement within 0–4 h before t0. Continuous variables are presented as mean ± standard deviation (SD) or median (interquartile range), as appropriate; categorical variables are presented as counts (percentages) [*n* (%)]. Welch *t* denotes the Welch two-sample t statistic, *χ*^2^ denotes the Pearson chi-square statistic, *Z* denotes the standardized Mann–Whitney *U*-statistic, and *p* denotes the two-sided *p*-value. SCr, serum creatinine; MAP, mean arterial pressure; PaO₂/FiO₂, ratio of arterial partial pressure of oxygen to fraction of inspired oxygen. Protocol adherence was calculated conservatively; all tests were two-sided.

### 3.3 Model development and nomogram

The predefined six predictors were entered into the model simultaneously. Higher SOFA score, sepsis-associated AKI, and vasoactive drug use were associated with a lower probability of successful 7-day liberation, whereas greater cumulative urine output during the 24 h before discontinuation, larger relative SCr decline during the last 24 h, and higher 24-h time-weighted mean MAP were associated with a higher probability of success. The uniform bootstrap shrinkage factor was 0.94; the shrunken slopes and re-estimated intercept constituted the final equation (Table 3).

**Table 3:** Multivariable logistic model for predicting successful 7-day liberation from continuous renal replacement therapy (CRRT).

Variable	Unshrunken *β*	Final shrunken *β*	SE (unshrunken)	OR (95% CI, unshrunken)	*p*-value (unshrunken)
SOFA score (per 1-point increase)	−0.182	−0.171	0.047	0.833 (0.760–0.914)	<0.001
Sepsis-associated AKI (yes vs. no)	−0.712	−0.669	0.302	0.491 (0.272–0.887)	0.018
24 h cumulative urine output (per 100 mL)	0.113	0.107	0.022	1.120 (1.072–1.170)	<0.001
SCr decline during the last 24 h (per 1%)	0.041	0.039	0.009	1.042 (1.024–1.061)	<0.001
24 h time-weighted mean MAP (per 1 mmHg)	0.053	0.050	0.014	1.055 (1.027–1.084)	<0.001
Vasoactive drug use (yes vs. no)	−1.027	−0.965	0.317	0.358 (0.192–0.667)	0.001
Intercept	−2.789	−2.620*	—	—	—

**Figure 2:**
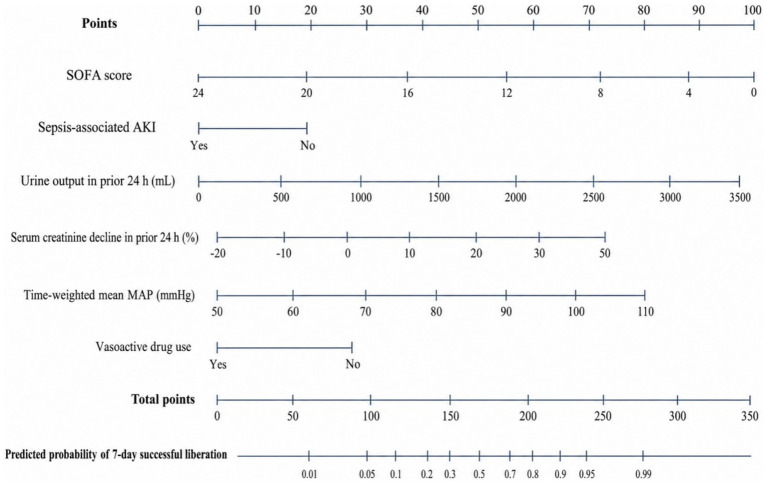
Standard nomogram for predicting successful 7-day liberation from continuous renal replacement therapy (CRRT). Points corresponding to Sequential Organ Failure Assessment (SOFA) score, sepsis-associated acute kidney injury (AKI), cumulative urine output during the 24 h before discontinuation, relative serum creatinine (SCr) decline during the last 24 h, 24-h time-weighted mean arterial pressure (MAP), and vasoactive drug use are summed and mapped to the predicted probability. Each axis is scaled according to the predictor range and coefficient in the final equation.

*β*, logistic regression coefficient; SE, standard error; OR, odds ratio; CI, confidence interval; P, two-sided *p*-value; vs, versus. CRRT, continuous renal replacement therapy; SOFA, Sequential Organ Failure Assessment; AKI, acute kidney injury; SCr, serum creatinine; MAP, mean arterial pressure. OR, 95% CI, and *p*-values are from the unshrunken inferential model. The final prediction equation uses uniformly shrunken slopes and a re-estimated intercept; this intercept is used only for probability calculation.

logit(*P*) = −2.620 − 0.171 × SOFA − 0.669 × Sepsis + 0.107 × (UO24/100) + 0.039 × SCrDecline + 0.050 × MAP − 0.965 × Vasoactive; *P* = exp.[logit(*P*)]/{1 + exp.[logit(*P*)]}. Sepsis and Vasoactive were coded as yes = 1 and no = 0. UO24 is expressed in mL and entered per 100 mL; SCrDecline is expressed as a percentage; MAP is expressed in mmHg. Based on this final equation, the points for each predictor and the total score can be mapped to the probability of successful 7-day liberation (Figure 2).

**Figure 3:**
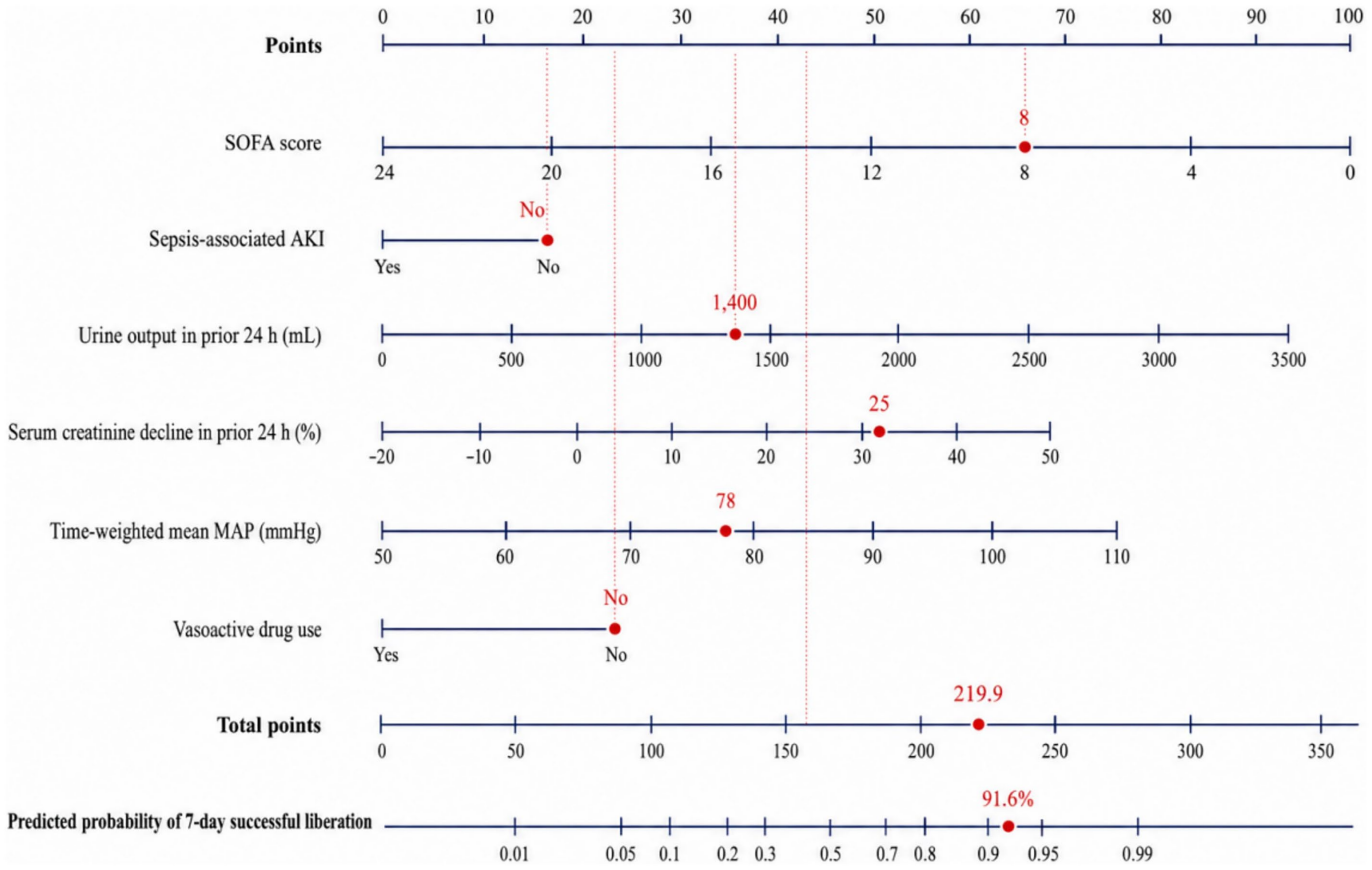
Clinical nomogram example for predicting successful 7-day liberation from continuous renal replacement therapy (CRRT). Red dots and light-red vertical lines indicate each variable value and its corresponding point score; red dots also indicate the total score and predicted probability. The example patient had a Sequential Organ Failure Assessment (SOFA) score of 8, no sepsis-associated acute kidney injury (AKI), cumulative urine output of 1,400 mL during the 24 h before discontinuation, a 25% relative serum creatinine (SCr) decline during the last 24 h, a 24-h time-weighted mean arterial pressure (MAP) of 78 mmHg, and no vasoactive drug use; the final equation predicted a 91.6% probability of successful 7-day liberation.

For example, for a patient with a SOFA score of 8, no sepsis-associated AKI, cumulative urine output of 1,400 mL during the 24 h before discontinuation, a 25% relative SCr decline during the last 24 h, a 24-h time-weighted mean MAP of 78 mmHg, and no vasoactive drug use, the final equation predicts a 91.6% probability of successful 7-day liberation (Figure 3).

### 3.4 Model performance

**Figure 4:**
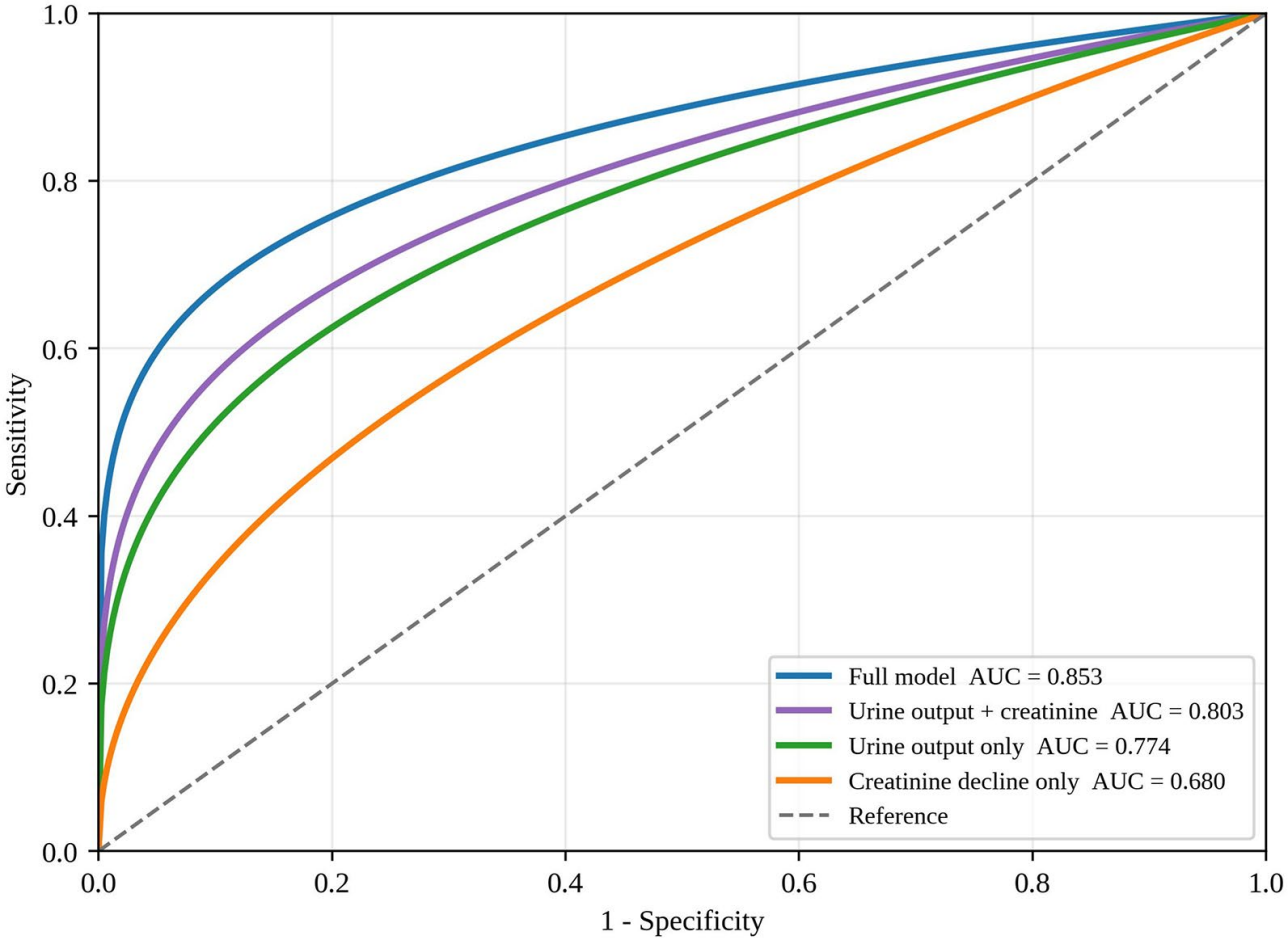
Receiver operating characteristic (ROC) curve comparison in the validation sample. The full model, urine output alone model, serum creatinine (SCr) decline alone model, and urine output plus SCr model were evaluated in the same validation sample. The area under the receiver operating characteristic curve (AUC) indicates discrimination; the dashed line indicates no discriminative ability.

**Figure 5:**
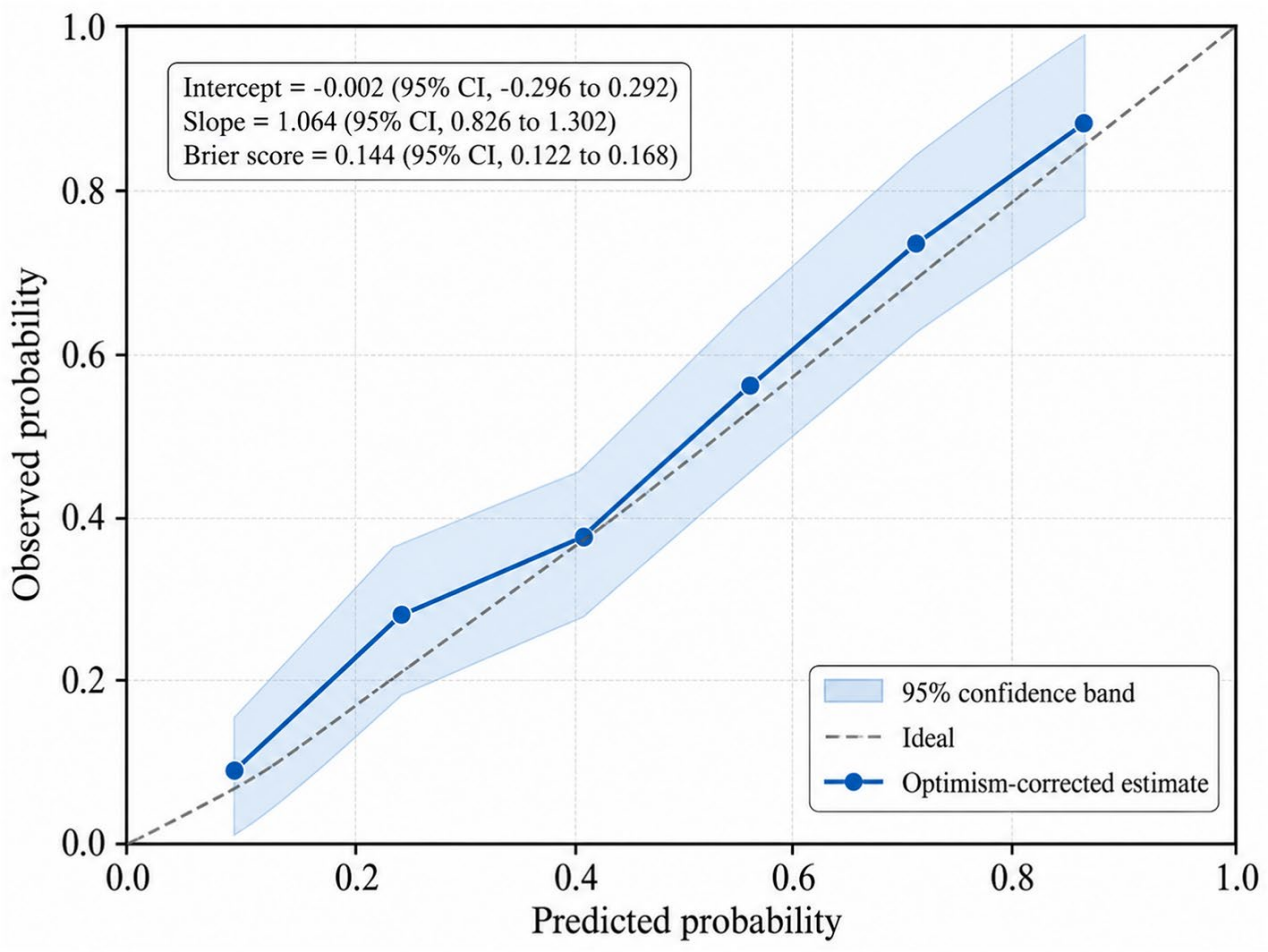
Bootstrap calibration curve in the development sample. The dashed line indicates ideal calibration; the solid line and points indicate the optimism-corrected estimate; the shaded area indicates the 95% confidence interval (CI).

**Figure 6:**
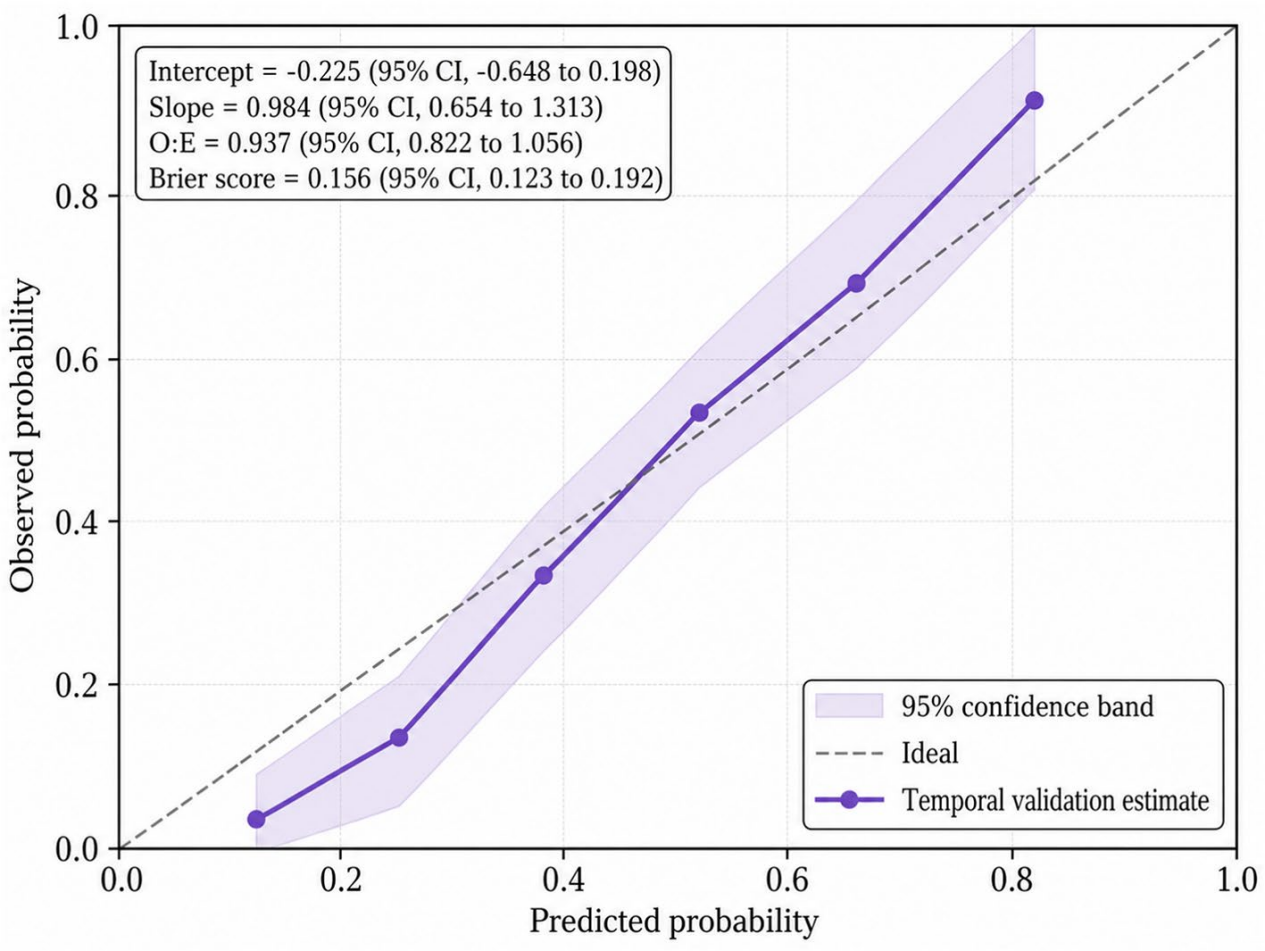
Calibration curve in the validation sample. The dashed line indicates ideal calibration; the solid line and points indicate observed results; the shaded area indicates the 95% confidence interval (CI). Mild overprediction was present at low predicted probabilities. O: E, observed-to-expected ratio.

**Figure 7:**
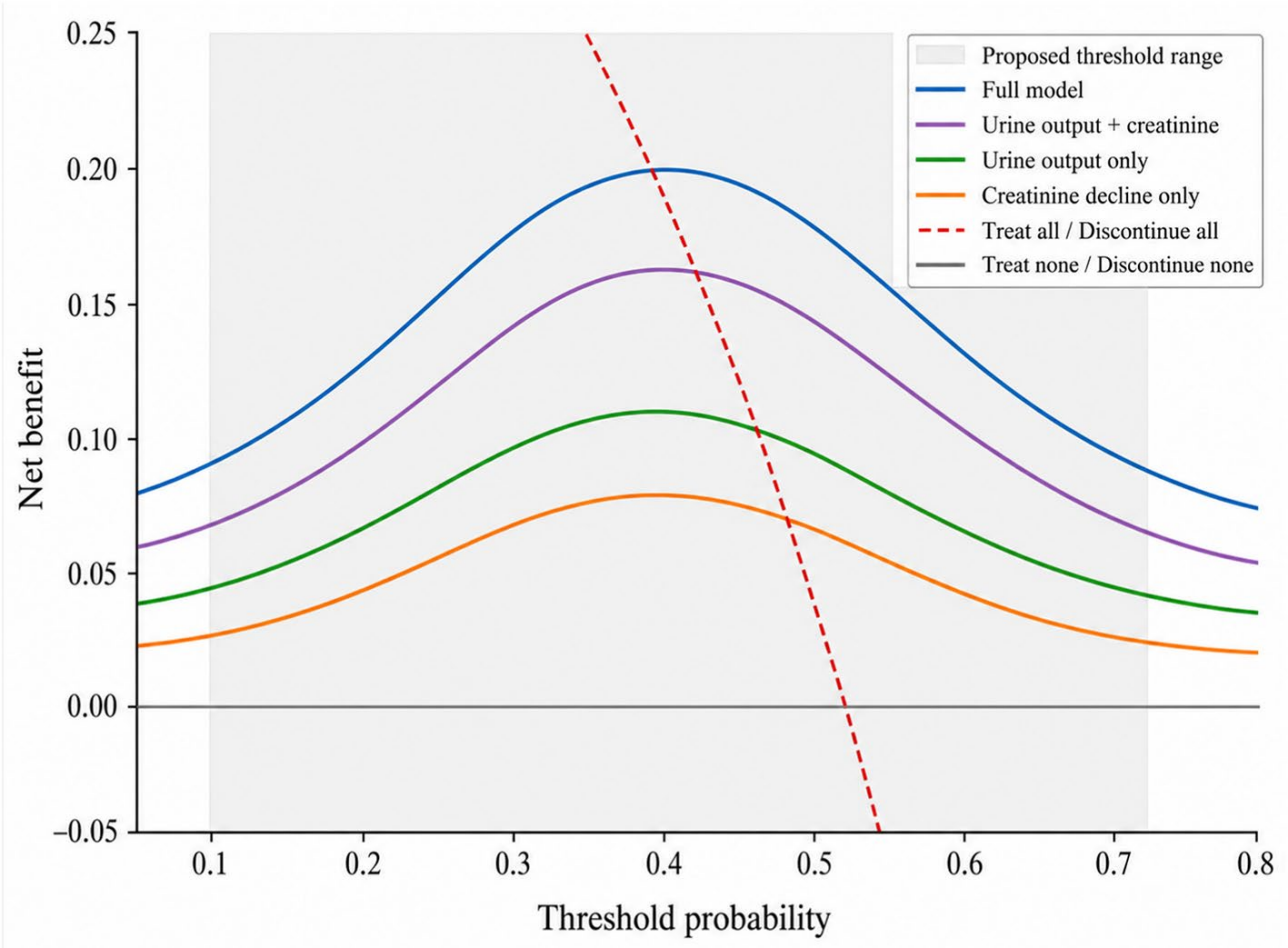
Decision curve analysis (DCA) in the validation sample. Net benefit was compared among the full model, urine output alone model, serum creatinine (SCr) decline alone model, urine output plus SCr model, and the strategies of discontinuing continuous renal replacement therapy (CRRT) in all patients and continuing CRRT in all patients. The light-gray area indicates the predefined evaluation range of 0.25–0.75. Between approximately 0.43 and 0.75, the full model provided greater net benefit than both default strategies.

In the development sample, the apparent AUC decreased only slightly after bootstrap optimism correction; in the subsequent-year validation sample, model discrimination remained good. In both samples, Brier score, calibration intercept, and calibration slope were generally close to ideal values, and the validation O: E ratio was close to 1. When the threshold determined in the development sample was applied directly to the validation sample, sensitivity exceeded specificity, and positive and negative predictive values were 77.11 and 80.00%, respectively. The Hosmer–Lemeshow test was not statistically significant in the development sample (*p* > 0.05) but was significant in the validation sample (*p* = 0.044); the calibration curve suggested mild overprediction at low predicted probabilities. Decision curve analysis (DCA) assessed net benefit across the predefined threshold probability range of 0.25–0.75; between approximately 0.43 and 0.75, the full model provided greater net benefit than the two default strategies of discontinuing CRRT in all patients and continuing CRRT in all patients (Table 4; Figures 4–7).

**Table 4:** Model performance in the development cohort and temporal validation cohort.

Performance measure	Development cohort	Temporal validation cohort	95% CI/*p*-value	Notes
Sample size (success/failure)	304 (165/139)	143 (76/67)	—	Temporal split by discontinuation date
AUC	0.870	0.853	Development: 0.830–0.907; validation: 0.785–0.913	Development: apparent performance; validation: final equation
Bootstrap optimism	0.010	—	0.004–0.020	1,000 resamples
Optimism-corrected AUC	0.860	—	0.817–0.900	Development cohort
Fixed threshold	0.529	0.529	—	Development-cohort Youden index
Sensitivity	—	84.21%	74.40–90.73%	64/76
Specificity	—	71.64%	59.91–81.03%	48/67
Positive predictive value	—	77.11%	66.99–84.83%	64/83
Negative predictive value	—	80.00%	68.22–88.17%	48/60
Brier score	0.144	0.156	Development: 0.122–0.168; validation: 0.123–0.192	Lower is better
Calibration intercept	−0.002	−0.225	Development: −0.296–0.292; validation: −0.648–0.198	Ideal value: 0
Calibration slope	1.064	0.984	Development: 0.826–1.302; validation: 0.654–1.313	Ideal value: 1
O:E	—	0.937	0.822–1.056	Ideal value: 1
Hosmer–Lemeshow	*χ*^2^ = 11.412, *p* = 0.179	*χ*^2^ = 15.915, *P* = 0.044	—	Interpret together with calibration curve
Threshold probability range in which the full model outperformed both default strategies	—	Approximately 0.43–0.75	—	Predefined evaluation range: 0.25–0.75

*n*, sample size; AUC, area under the receiver operating characteristic curve; CI, confidence interval. Bootstrap denotes resampling with replacement. Optimism was the difference between apparent performance in the development data and test performance in bootstrap resamples. The fixed threshold was determined by the maximum Youden index in the development cohort; the Youden index is defined as sensitivity + specificity − 1. Sensitivity is the proportion of truly successfully liberated patients classified as successful; specificity is the proportion of patients with liberation failure classified as failed; positive predictive value is the proportion of predicted successes who were truly successful; negative predictive value is the proportion of predicted failures who truly failed. The Brier score is the mean squared difference between predicted probabilities and observed outcomes, with lower values indicating smaller overall prediction error. The ideal calibration intercept is 0, and the ideal calibration slope is 1. O:E, observed-to-expected ratio; CRRT, continuous renal replacement therapy; DCA, decision curve analysis; *χ*^2^, chi-square statistic; *P*, two-sided *p*-value. The threshold derived from the development cohort was applied unchanged to the temporal validation cohort. The 95% CI values for calibration measures were obtained from 2,000 patient-level bootstrap resamples, with imputation repeated within each resample. Decision curves were evaluated across the predefined threshold probability range of 0.25–0.75; the full model exceeded both default strategies between approximately 0.43 and 0.75. “Discontinue CRRT in all patients” denotes discontinuing CRRT for every patient, and “continue CRRT in all patients” denotes continuing CRRT for every patient; both are default comparator strategies in DCA.

### 3.5 Comparative models, sensitivity analyses, and subgroup analyses

In the validation sample, the AUC of the full model was higher than that of the urine output alone model and the SCr decline alone model (*p* = 0.024 and *p* < 0.001, respectively), whereas the difference from the urine output plus SCr model was not statistically significant (*p* = 0.094). After removal of urine output and SCr, discrimination decreased markedly. The basic clinical model containing only sepsis-associated AKI and CKD had an AUC of 0.651 (*p* < 0.001). Discrimination was higher when the 72-h outcome was used than when the 7-day primary outcome was used; when failure was restricted to KRT restart among 7-day survivors, model performance was similar to the primary analysis. The AUC values in the protocol-adherent subset, complete-case subset, model substituting absolute SCr decline for relative SCr decline, and fixed median/mode imputation analysis were all close to the primary model, and none of the differences was statistically significant (all *p* > 0.05) (Table 5).

**Table 5:** Comparative models and sensitivity analyses.

Analysis/model	Validation sample	AUC (95% CI)	ΔAUC versus full model (95% CI)	*p*-value
Full model	*n* = 143	0.853 (0.785–0.913)	Reference	—
24 h urine output only	*n* = 143	0.774 (0.694–0.845)	0.079 (0.010–0.155)	0.024
SCr decline only	*n* = 143	0.680 (0.589–0.766)	0.173 (0.083–0.265)	<0.001
Urine output + SCr	*n* = 143	0.803 (0.727–0.872)	0.050 (−0.010–0.111)	0.094
Excluding urine output and SCr	*n* = 143	0.765 (0.684–0.840)	0.087 (0.031–0.147)	0.002
Basic clinical model: sepsis-associated AKI + CKD	*n* = 143	0.651 (0.557–0.739)	0.202 (0.112–0.294)	<0.001
72 h outcome	*n* = 143; 85 successes	0.891 (0.830–0.942)	Different outcome; descriptive only	—
7-day survivors: KRT restart outcome	*n* = 138; 76 successes	0.846 (0.775–0.909)	Different validation sample; descriptive only	—
Protocol-adherent validation subset	*n* = 129	0.849 (0.777–0.910)	Different validation sample; descriptive only	—
Complete-case validation subset	*n* = 135	0.847 (0.779–0.907)	Different validation sample; descriptive only	—
Absolute SCr decline replacing relative decline	*n* = 143	0.848 (0.779–0.907)	0.005 (−0.011–0.022)	0.548
Fixed median/mode imputation	*n* = 143	0.850 (0.781–0.910)	0.003 (−0.013–0.020)	0.706

*n*, sample size; AUC, area under the receiver operating characteristic curve; ΔAUC, difference between the AUC of the full model and that of the comparator model; CI, confidence interval. Paired bootstrap denotes resampling of the same validation patients with replacement and calculation of the AUC difference. *p* denotes the two-sided *p*-value. SCr, serum creatinine; AKI, acute kidney injury; CKD, chronic kidney disease; KRT, kidney replacement therapy. Analyses labeled “different outcome” or “different validation sample” are presented descriptively only and were not formally compared with the primary model.

Across all 603 planned discontinuation attempts, 344 met the definition of successful 7-day liberation. In the patient-clustered GEE analysis, the effect directions of all six predictors were consistent with those in the primary model based on first attempts (Supplementary Table S3).

**Figure 8:**
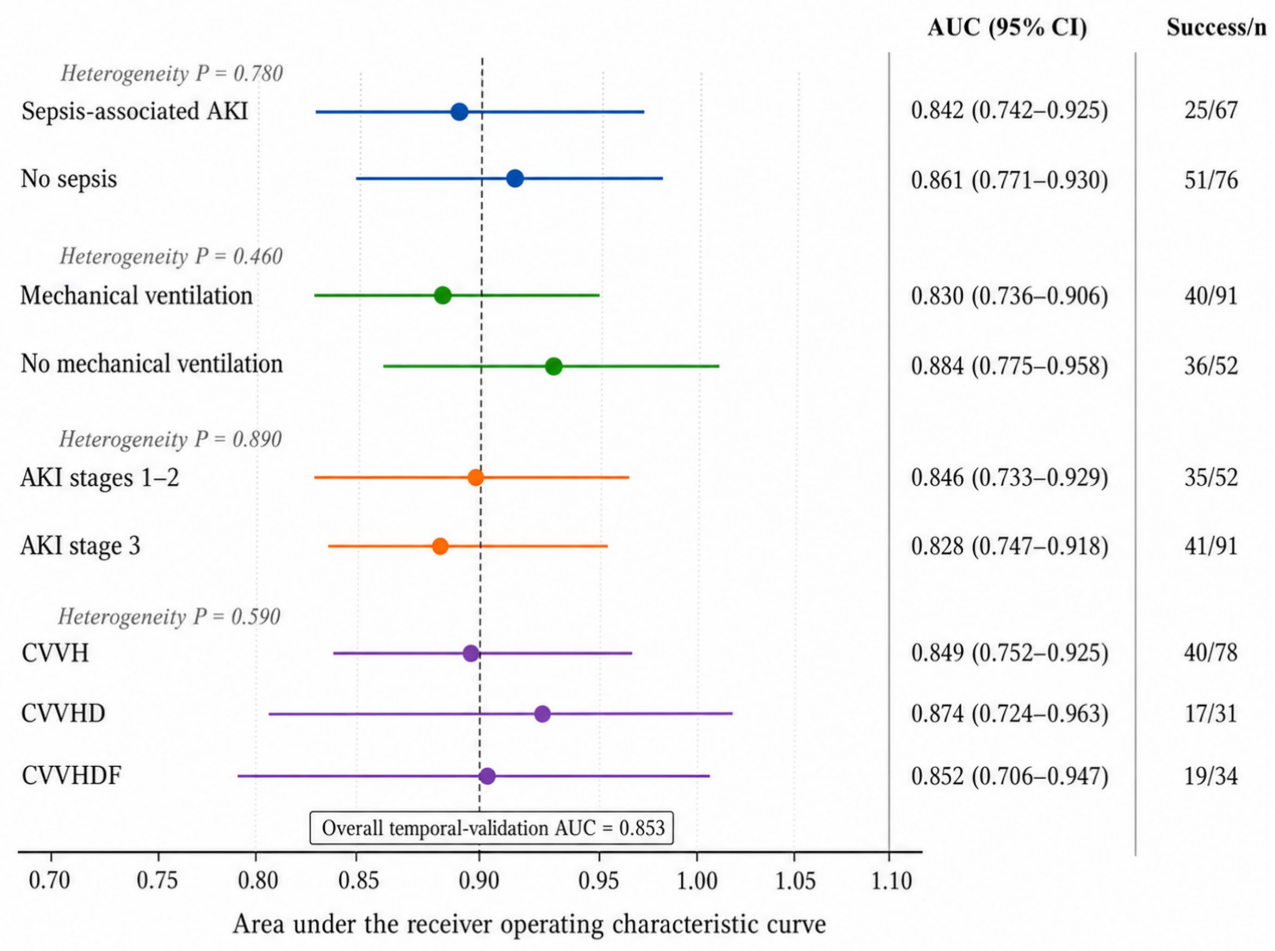
Forest plot of area under the receiver operating characteristic curve (AUC) in exploratory subgroups of the validation sample. Points indicate AUC, horizontal lines indicate 95% confidence intervals (CI), colors distinguish predefined strata, and the vertical dashed line indicates the overall temporal validation AUC. Heterogeneity tests were not adjusted for multiple comparisons. AKI, acute kidney injury; CRRT, continuous renal replacement therapy; CVVH, continuous venovenous hemofiltration; CVVHD, continuous venovenous hemodialysis; CVVHDF, continuous venovenous hemodiafiltration.

Across the predefined subgroups in the validation sample, the model maintained moderate-to-good discrimination. Heterogeneity tests stratified by sepsis, mechanical ventilation, AKI stage, and CRRT mode all had *p*-values >0.05. These results indicate only that no clear performance heterogeneity was detected in this validation sample (Supplementary Table S4; Figure 8).

### 3.6 Secondary outcomes

The failure group had a higher 28-day all-cause mortality rate. The numbers of 28-day and 90-day survivors served as the denominators for analyses of renal recovery and chronic dialysis dependence, respectively. Among 28-day survivors, the success group had a higher complete renal recovery rate and lower rates of partial recovery and no recovery. Among 90-day survivors, chronic dialysis dependence was more common in the failure group (all *p* < 0.001) (Table 6).

**Table 6:** Comparison of secondary outcomes between groups.

Outcome	Success group	Failure group	Statistic	*p*-value
28-day all-cause mortality	15/241 (6.22)	72/206 (34.95)	*χ*^2^ = 58.474	<0.001
28-day survivors	226	134	—	—
Complete renal recovery*	158/226 (69.91)	40/134 (29.85)		
Partial renal recovery*	54/226 (23.89)	47/134 (35.07)	*χ*^2^ = 69.702	<0.001
No recovery*	14/226 (6.19)	47/134 (35.07)		
90-day survivors	218	114	—	—
90-day chronic dialysis dependence†	12/218 (5.50)	40/114 (35.09)	*χ*^2^ = 49.594	<0.001

Categorical variables are presented as counts/corresponding denominators (percentages) [*n*/*N* (%)]. *χ*^2^ denotes the Pearson chi-square statistic, and P denotes the two-sided *p*-value. *Renal recovery at 28 days was assessed among 28-day survivors. ^†^Chronic dialysis dependence at 90 days was assessed among 90-day survivors. Complete recovery was defined as SCr ≤1.5 times the baseline value; partial recovery was defined as not meeting the criterion for complete recovery and having an SCr decrease of ≥50% from CRRT initiation; no recovery was defined as an SCr decrease of <50% from CRRT initiation. SCr, serum creatinine; CRRT, continuous renal replacement therapy.

## 4 Discussion

The main findings were as follows. Among 447 patients with AKI undergoing a first planned CRRT discontinuation attempt, the 7-day successful liberation rate was 53.91%. The six-variable model maintained good discrimination after bootstrap optimism correction in the development sample and in the subsequent-year validation sample. Overall calibration in the validation sample was acceptable, although mild overprediction occurred at low predicted probabilities. The full model outperformed urine output alone and SCr decline alone, but its incremental discrimination over the urine output plus SCr model was limited. Discrimination decreased markedly after removing urine output and SCr, suggesting that dynamic pre-discontinuation kidney function information remains a core component of CRRT liberation risk stratification.

Current evidence on renal replacement therapy (RRT) discontinuation suggests that urine output, creatinine clearance, urinary creatinine, NGAL, CRRT duration, and ICU length of stay are associated with discontinuation success, although outcome definitions, measurement windows, and candidate predictors remain substantially heterogeneous (21). The temporal validation AUC in the present study was 0.853, within the moderate-to-good discrimination range reported by recent multivariable and machine learning models for CRRT liberation; compared with some high-dimensional models, the six-variable model retained a simpler and clinically interpretable structure (22). Recent data on adult CRRT transition similarly suggest that urine output, kidney function measures, circulatory stability, and respiratory support status are closely related to successful transition (23). Substantial variation in CRRT prescription, anticoagulation strategy, and operational parameters across critical care settings supports the need to preserve local workflow context when interpreting model results (24). Data across age groups also show that urine output and volume status are associated with CRRT liberation, suggesting that these physiological signals have a consistent clinical direction (25).

The primary outcome was defined as survival without any form of KRT for 7 consecutive days after CRRT discontinuation. Compared with the 72-h outcome, the 7-day outcome classified 27 patients with KRT restart or death on days 4–7 as failures, thereby more fully capturing early delayed failure. Liberation failure was determined only by actual KRT restart or all-cause death within 7 days; post-discontinuation SCr elevation or reduced urine output was treated as a signal for clinical reassessment, which helps reduce direct overlap between predictors and the outcome definition.

Higher SOFA score and sepsis-associated AKI were associated with a lower probability of successful liberation. Sepsis-associated AKI involves inflammatory dysregulation, microcirculatory impairment, endothelial injury, and metabolic reprogramming, mechanisms that may delay renal and systemic organ recovery (26). Critically ill patients with sepsis-associated AKI are substantially heterogeneous in timing, trajectory, treatment requirements, and prognosis, consistent with the direction observed in this study whereby sepsis-associated AKI lowered the probability of successful liberation (27). CKD stages 1–4 were more common in the failure group, suggesting that lower renal reserve may increase the risk of incomplete recovery and adverse long-term renal outcomes after AKI (28). These variables reflect the systemic disease context of the patient, and their effect estimates are appropriate for prediction.

Urine output, MAP, and vasoactive drug use during the 24 h before discontinuation reflect volume-handling capacity and circulatory stability. Intradialytic hypotension after transition from CRRT to intermittent hemodialysis is associated with in-hospital mortality and delayed liberation from RRT, highlighting the importance of hemodynamic tolerance during treatment de-escalation (29). Supportive care for AKI emphasizes integrated assessment of volume management, blood pressure support, electrolytes, and acid–base balance, consistent with the model’s integration of kidney function, circulatory status, and organ function into risk estimation (30). Thus, the clinical value of this model does not depend on a single indicator; rather, it integrates multidimensional information available at the bedside before discontinuation into an individualized probability of 7-day liberation.

A decline in SCr is expected while patients receive CRRT. This study used the relative SCr change within a fixed 24-h window before discontinuation and observed no significant difference in delivered dialysate/replacement fluid flow rate between groups. This measure reflects the net effects of extracorporeal clearance, endogenous generation, volume of distribution, and residual kidney function. Conventional SCr and urine output have temporal lag and specificity limitations in AKI assessment and should be interpreted in the context of treatment background and clinical status (31). Novel biomarkers may provide complementary information on tubular injury, cellular stress, or filtration function, but their clinical translation still depends on standardized sampling, threshold validation, and reproducibility assessment (32). Longitudinal NGAL and cystatin C trajectories in mixed ICU populations can show substantial interindividual heterogeneity, suggesting that a single measurement may not adequately represent the recovery process (33). This cohort did not have a standardized dynamic pre-discontinuation sampling protocol; therefore, these biomarkers were not included in the final model.

DCA showed that the full model provided higher net benefit than the two default strategies of discontinuing CRRT in all patients and continuing CRRT in all patients over threshold probabilities of approximately 0.43–0.75. This range corresponds to clinical situations in which clinicians have moderate uncertainty about whether a patient can be successfully liberated from CRRT: the patient has met the discontinuation checklist, but urine output, SCr, or bedside judgment alone remains insufficient to quantify the probability of 7-day liberation. Within this threshold range, the model may help clinicians weigh the risk of KRT restart against the trade-off of prolonged CRRT exposure. DCA can quantify the theoretical clinical value of a prediction model across different threshold probabilities, but patient-level real-world benefit still requires evaluation in prospective impact studies (34).

From the standpoint of prediction model evaluation, discrimination, calibration, overall accuracy, and clinical utility should be reported together, because models with similar AUC values may differ in calibration performance and decision value (35). This study used calendar-time splitting for temporal validation within the same center, providing an initial assessment of model stability in later-year data. Because CRRT prescriptions, discontinuation practices, KRT restart thresholds, and patient populations may differ across institutions, geographical external validation should be performed before application in other healthcare settings, and the need for local recalibration should be assessed using calibration intercept, calibration slope, and the O: E ratio (36). External validation studies should also ensure a sufficient number of events and adequate estimation precision to reliably assess calibration and clinical utility (37). If the model is embedded in the electronic medical record or bedside decision-support system in the future, clinicians’ acceptance of model outputs, explanation methods, and workflow integration will also need to be evaluated (38).

This study has four main limitations. First, the single-center retrospective design, inclusion only of patients who reached a planned discontinuation attempt, and restriction of the primary analysis to first attempts may introduce selection bias and residual confounding; the repeated-attempt GEE analysis cannot fully eliminate these issues. Second, urine output, SCr, MAP, vasoactive drug use, and SOFA score are related to the local discontinuation workflow, and KRT restart was determined by integrated clinical judgment; therefore, both the model and outcome are workflow-dependent. Combining death within 7 days and KRT restart into a composite failure outcome may also obscure distinct event mechanisms. Third, validation was limited to a subsequent temporal cohort from the same center. Mild calibration deviation was observed at low predicted probabilities, and the validation, subgroup, and repeated-attempt sample sizes were limited. Fourth, dynamic pre-discontinuation cystatin C, NGAL, and TIMP-2·IGFBP7 were not collected using standardized procedures, and data on race/ethnicity, socioeconomic status, and healthcare access were insufficient, limiting full assessment of biomarker incremental value and model fairness. After PROBAST assessment, the overall risk of bias for model development and temporal validation was high.

## 5 Conclusion

We developed a model to estimate the probability of successful 7-day liberation from CRRT and temporally validated it in a subsequent cohort from the same center. The model provides reproducible individualized risk estimates and adds information beyond urine output or SCr alone. The model is intended for adjunctive risk stratification after standard CRRT discontinuation criteria have been fulfilled and the discontinuation checklist has been completed and should not replace clinical judgment; clinical decisions should integrate the predicted probability with volume status, electrolytes, acid–base balance, hemodynamic stability, and the patient’s overall goals of care. The cross-center performance of the final equation, the need for local recalibration, and its effects on clinical decisions and patient outcomes require evaluation in prospective multicenter studies.

## Data Availability

The original contributions presented in the study are included in the article/Supplementary material, further inquiries can be directed to the corresponding author.
