# Supplementary material for: Prediction model for successful liberation from continuous renal replacement therapy in patients with acute kidney injury: development and temporal validation
Source: Front Med (Lausanne). 2026 Jul 30;13:1882567. doi: 10.3389/fmed.2026.1882567 (PMC13470257; doi:10.3389/fmed.2026.1882567)
Supplement: Supplementary file 1 [file Table_1.DOCX]

# **Supplementary Materials**

Supplementary Table 1. Number and proportion of missing values for all collected variables

| Variable | Number missing | Missing proportion |
| --- | --- | --- |
| Age | 0 | 0.00% |
| Sex | 0 | 0.00% |
| BMI | 9 | 2.01% |
| Hypertension | 0 | 0.00% |
| Diabetes mellitus | 0 | 0.00% |
| Chronic heart failure | 0 | 0.00% |
| Chronic liver disease | 0 | 0.00% |
| Malignancy | 0 | 0.00% |
| Surgery within 30 days | 0 | 0.00% |
| CKD stages 1–4 | 0 | 0.00% |
| APACHE II | 4 | 0.89% |
| SOFA | 2 | 0.45% |
| AKI stage | 0 | 0.00% |
| AKI etiology | 0 | 0.00% |
| Baseline SCr | 18 | 4.03% |
| Urine output at ICU admission | 11 | 2.46% |
| Urine output during 24 h before CRRT initiation | 14 | 3.13% |
| SCr at CRRT initiation | 3 | 0.67% |
| BUN | 5 | 1.12% |
| Cystatin C | 37 | 8.28% |
| Serum potassium | 2 | 0.45% |
| Serum sodium | 2 | 0.45% |
| Serum chloride | 3 | 0.67% |
| Serum calcium | 6 | 1.34% |
| Serum phosphorus | 8 | 1.79% |
| pH | 4 | 0.89% |
| HCO₃⁻ | 6 | 1.34% |
| Lactate | 12 | 2.68% |
| White blood cell count | 4 | 0.89% |
| Hemoglobin | 3 | 0.67% |
| Platelet count | 7 | 1.57% |
| ALT | 9 | 2.01% |
| AST | 9 | 2.01% |
| Total bilirubin | 10 | 2.24% |
| PT | 8 | 1.79% |
| APTT | 8 | 1.79% |
| INR | 8 | 1.79% |
| Albumin | 5 | 1.12% |
| CRP | 16 | 3.58% |
| PCT | 24 | 5.37% |
| BNP | 41 | 9.17% |
| CRRT mode | 0 | 0.00% |
| Anticoagulation strategy | 0 | 0.00% |
| CRRT duration | 0 | 0.00% |
| Total dialysate/replacement fluid volume | 12 | 2.68% |
| Net ultrafiltration rate | 5 | 1.12% |
| Blood flow rate | 4 | 0.89% |
| Dialysate/replacement fluid flow rate | 6 | 1.34% |
| Number of filter replacements | 0 | 0.00% |
| Protocol adherence | 15 | 3.36% |
| Time from criteria fulfillment to treatment discontinuation | 15 | 3.36% |
| Mean hourly urine output | 7 | 1.57% |
| Urine output before discontinuation during 24 h | 7 | 1.57% |
| 24 h fluid intake | 8 | 1.79% |
| 24 h fluid output | 13 | 2.91% |
| 24 h fluid balance | 13 | 2.91% |
| Relative SCr decline | 11 | 2.46% |
| Absolute SCr decline | 11 | 2.46% |
| Relative BUN decline | 10 | 2.24% |
| Absolute BUN decline | 10 | 2.24% |
| 24 h time-weighted mean MAP | 5 | 1.12% |
| Vasoactive drug use | 0 | 0.00% |
| Norepinephrine use | 0 | 0.00% |
| Norepinephrine dose | 0 | 0.00% |
| Dopamine use | 0 | 0.00% |
| Epinephrine use | 0 | 0.00% |
| Vasopressin use | 0 | 0.00% |
| Duration of vasoactive drug use | 0 | 0.00% |
| Mechanical ventilation | 0 | 0.00% |
| PaO₂/FiO₂ | 19 | 4.25% |
| ECMO | 0 | 0.00% |
| IABP | 0 | 0.00% |
| Primary outcome | 0 | 0.00% |
| 28-day all-cause mortality | 0 | 0.00% |
| 28-day renal recovery | 0 | 0.00% |
| 90-day survival status | 0 | 0.00% |
| 90-day chronic dialysis dependence | 0 | 0.00% |

Note: Missing proportion = number of missing values / 447 × 100%. This table reports only missing frequencies and proportions and does not present between-group inferential tests. Imputation was performed separately in the development and temporal validation cohorts; the primary outcome and follow-up outcomes had no missing data. BMI, body mass index; CKD, chronic kidney disease; APACHE II, Acute Physiology and Chronic Health Evaluation II; SOFA, Sequential Organ Failure Assessment; AKI, acute kidney injury; SCr, serum creatinine; ICU, intensive care unit; CRRT, continuous renal replacement therapy; BUN, blood urea nitrogen; HCO₃⁻, bicarbonate; ALT, alanine aminotransferase; AST, aspartate aminotransferase; PT, prothrombin time; APTT, activated partial thromboplastin time; INR, international normalized ratio; CRP, C-reactive protein; PCT, procalcitonin; BNP, B-type natriuretic peptide; MAP, mean arterial pressure; PaO₂/FiO₂, ratio of arterial partial pressure of oxygen to fraction of inspired oxygen; ECMO, extracorporeal membrane oxygenation; IABP, intra-aortic balloon pump.

Supplementary Table 2. Comparison of key characteristics between the development cohort and temporal validation cohort

| Variable | Development cohort (*n* = 304) | Temporal validation cohort (*n* = 143) | Statistic | *P* value |
| --- | --- | --- | --- | --- |
| Sample size | 304 | 143 | — | — |
| 7-day success | 165 (54.28) | 76 (53.15) | *χ²* = 0.050 | 0.823 |
| Age (years) | 61.9 ± 14.2 | 62.5 ± 14.5 | Welch *t* = −0.411 | 0.682 |
| Male sex | 188 (61.84) | 88 (61.54) | *χ²* = 0.004 | 0.951 |
| CKD stages 1–4 | 66 (21.71) | 32 (22.38) | *χ²* = 0.025 | 0.874 |
| Sepsis-associated AKI | 142 (46.71) | 67 (46.85) | *χ²* = 0.001 | 0.978 |
| SOFA score | 9.78 ± 3.54 | 9.92 ± 3.48 | Welch *t* = −0.395 | 0.693 |
| Cumulative urine output during 24 h before discontinuation (mL) | 930 (500, 1500) | 915 (490, 1490) | *Z* = −0.327 | 0.744 |
| Relative SCr decline during the last 24 h (%) | 18.4 ± 17.1 | 18.0 ± 16.8 | Welch *t* = 0.233 | 0.816 |
| 24 h time-weighted mean MAP (mmHg) | 72.9 ± 12.0 | 73.1 ± 11.8 | Welch *t* = −0.166 | 0.868 |
| Vasoactive drug use | 191 (62.83) | 90 (62.94) | *χ²* < 0.001 | 0.982 |
| CRRT mode: CVVH/CVVHD/CVVHDF | 163/68/73 | 77/32/34 | *χ²* = 0.003 | 0.998 |

Note: Continuous variables are presented as mean ± standard deviation (SD) or median (interquartile range), as appropriate; categorical variables are presented as counts (percentages) [*n* (%)]. Welch *t* denotes the Welch two-sample *t* statistic, *χ²* denotes the Pearson chi-square statistic, *Z* denotes the standardized Mann–Whitney *U* statistic, and *P* denotes the two-sided *P* value. CKD, chronic kidney disease; AKI, acute kidney injury; SOFA, Sequential Organ Failure Assessment; SCr, serum creatinine; MAP, mean arterial pressure; CRRT, continuous renal replacement therapy; CVVH, continuous venovenous hemofiltration; CVVHD, continuous venovenous hemodialysis; CVVHDF, continuous venovenous hemodiafiltration.

Supplementary Table 3. GEE sensitivity analysis of all 603 discontinuation attempts

| Variable | GEE OR (95% CI) | *P* value |
| --- | --- | --- |
| SOFA score (per 1-point increase) | 0.857 (0.806–0.911) | <0.001 |
| Sepsis-associated AKI | 0.511 (0.337–0.774) | 0.002 |
| 24 h cumulative urine output (per 100 mL) | 1.121 (1.086–1.157) | <0.001 |
| SCr decline during the last 24 h (per 1%) | 1.044 (1.030–1.059) | <0.001 |
| 24 h time-weighted mean MAP (per 1 mmHg) | 1.048 (1.030–1.067) | <0.001 |
| Vasoactive drug use | 0.477 (0.312–0.729) | <0.001 |

Note: GEE, generalized estimating equations; OR, odds ratio; CI, confidence interval; SE, standard error; *P*, two-sided *P* value. The binomial model used a logit link function. SOFA, Sequential Organ Failure Assessment; AKI, acute kidney injury; SCr, serum creatinine; MAP, mean arterial pressure. Patients were used as the clustering unit, with an exchangeable working correlation structure and robust SE.

Supplementary Table 4. Exploratory subgroup analyses in the temporal validation cohort

| Subgroup | *n* | Number successful | AUC (95% CI) | Heterogeneity *P* value |
| --- | --- | --- | --- | --- |
| Sepsis-associated AKI | 67 | 25 | 0.842 (0.742–0.925) | 0.780 |
| No sepsis | 76 | 51 | 0.861 (0.771–0.930) | — |
| Mechanical ventilation | 91 | 40 | 0.830 (0.736–0.906) | 0.460 |
| No mechanical ventilation | 52 | 36 | 0.884 (0.775–0.958) | — |
| AKI stages 1–2 | 52 | 35 | 0.846 (0.733–0.929) | 0.820 |
| AKI stage 3 | 91 | 41 | 0.858 (0.767–0.928) | — |
| CVVH | 78 | 40 | 0.849 (0.752–0.925) | 0.910 |
| CVVHD | 31 | 17 | 0.874 (0.724–0.963) | — |
| CVVHDF | 34 | 19 | 0.852 (0.706–0.947) | — |

Note: *n*, subgroup sample size; AUC, area under the receiver operating characteristic curve; CI, confidence interval; *P*, two-sided *P* value for heterogeneity or global testing. Bootstrap denotes patient-level resampling with replacement. AKI, acute kidney injury; CRRT, continuous renal replacement therapy; CVVH, continuous venovenous hemofiltration; CVVHD, continuous venovenous hemodialysis; CVVHDF, continuous venovenous hemodiafiltration. For each two-level stratum, the *P* value in the first row is from the heterogeneity test comparing subgroup AUC values; for CRRT mode, the *P* value in the first row is from the global test. No adjustment was made for multiple comparisons.

Supplementary Table 5. Official TRIPOD checklist for model development and validation

| Item | Official checklist item | Location (minimum section heading) | Reporting status |
| --- | --- | --- | --- |
| 1 (D;V) | Identify the study as developing and/or validating a multivariable prediction model, the target population, and the outcome to be predicted in the title. | Title | Reported. |
| 2 (D;V) | Provide a summary of objectives, study design, setting, participants, sample size, predictors, outcome, statistical analysis, results, and conclusions. | Abstract | Reported. |
| 3a (D;V) | Explain the medical context, including diagnostic or prognostic nature, and rationale for developing or validating the multivariable prediction model, with references to existing models. | 1 Introduction | Reported. |
| 3b (D;V) | Specify the objectives, including whether the study describes model development, model validation, or both. | 1 Introduction | Reported. |
| 4a (D;V) | Describe the study design or source of data, such as randomized trial, cohort, or registry data; separately describe development and validation datasets, if applicable. | 2.1 Study Design and Participants | Reported. |
| 4b (D;V) | Specify the key study dates, including start of accrual, end of accrual, and end of follow-up, if applicable. | 2.1 Study Design and Participants | Reported. |
| 5a (D;V) | Specify key elements of the study setting, such as primary care, secondary care, or general population, including number and location of centers. | 2.1 Study Design and Participants | Reported. |
| 5b (D;V) | Describe participant eligibility criteria. | 2.1 Study Design and Participants | Reported. |
| 5c (D;V) | Give details of treatments received, if relevant. | 2.2 CRRT Protocol, Discontinuation Checklist, and KRT Restart | Reported. |
| 6a (D;V) | Clearly define the outcome predicted by the prediction model, including how and when it was assessed. | 2.3 Data Collection, Time Windows, and Outcomes | Reported. |
| 6b (D;V) | Report any blinding used for assessment of the outcome to be predicted. | 2.3 Data Collection, Time Windows, and Outcomes | Reported. |
| 7a (D;V) | Clearly define all predictors used in developing or validating the multivariable prediction model, including how and when they were measured. | 2.3 Data Collection, Time Windows, and Outcomes | Reported. |
| 7b (D;V) | Report any blinding used for assessment of predictors relative to the outcome and other predictors. | 2.3 Data Collection, Time Windows, and Outcomes | Reported. |
| 8 (D;V) | Explain how the study sample size was determined. | 2.4 Statistical Analysis | Reported. |
| 9 (D;V) | Describe how missing data were handled, such as complete-case analysis, single imputation, or multiple imputation, and provide details of the imputation method. | 2.4 Statistical Analysis; Supplementary Materials | Reported. |
| 10a (D) | Describe how predictors were handled in the analyses. | 2.4 Statistical Analysis | Reported. |
| 10b (D) | Specify the model type, all modeling procedures, including any predictor selection, and the method used for internal validation. | 2.4 Statistical Analysis | Reported. |
| 10c (V) | For validation, describe how predictions were calculated. | 2.4 Statistical Analysis | Reported. |
| 10d (D;V) | Specify all measures used to assess model performance and, if relevant, to compare multiple models. | 2.4 Statistical Analysis | Reported. |
| 10e (V) | If model updating was performed, describe any model updating arising from validation, such as recalibration. | 2.4 Statistical Analysis | Not applicable. |
| 11 (D;V) | If risk groups were created, provide details on how risk groups were created. | 2.4 Statistical Analysis | Not applicable. |
| 12 (V) | For validation, identify any differences from the development data in setting, eligibility criteria, outcome, and predictors. | 3.1 Patient Flow, Primary Outcome, and Baseline Characteristics; Supplementary Materials | Reported. |
| 13a (D;V) | Describe the flow of participants through the study, including numbers with and without the outcome and, if applicable, a summary of follow-up time. A diagram may be helpful. | 3.1 Patient Flow, Primary Outcome, and Baseline Characteristics | Reported. |
| 13b (D;V) | Describe participant characteristics, including basic demographics, clinical features, and available predictors, including the number of participants with missing data for predictors and outcome. | 3.1 Patient Flow, Primary Outcome, and Baseline Characteristics; Supplementary Materials | Reported. |
| 13c (V) | For validation, show a comparison with the development data of the distribution of important variables, including demographics, predictors, and outcome. | 3.1 Patient Flow, Primary Outcome, and Baseline Characteristics; Supplementary Materials | Reported. |
| 14a (D) | Specify the number of participants and outcome events in each analysis. | 3.1 Patient Flow, Primary Outcome, and Baseline Characteristics; 3.3 Model Development and Nomogram | Reported. |
| 14b (D) | If performed, report the unadjusted association between each candidate predictor and outcome. | 2.4 Statistical Analysis | Not applicable. |
| 15a (D) | Present the full prediction model to allow predictions for individuals, such as all regression coefficients and the model intercept or the baseline survival at a given time point. | 3.3 Model Development and Nomogram | Reported. |
| 15b (D) | Explain how to use the prediction model. | 3.3 Model Development and Nomogram | Reported. |
| 16 (D;V) | Report performance measures, including confidence intervals, for the prediction model. | 3.4 Model Performance | Reported. |
| 17 (V) | If model updating was performed, report the results of model updating, including model specification and model performance. | 3.4 Model Performance | Not applicable. |
| 18 (D;V) | Discuss any limitations of the study, such as nonrepresentative sample, few events per predictor, or missing data. | 4 Discussion | Reported. |
| 19a (V) | For validation, discuss the results with reference to performance in the development data and any other validation data. | 4 Discussion | Reported. |
| 19b (D;V) | Provide an overall interpretation of results, considering objectives, limitations, results from similar studies, and other relevant evidence. | 4 Discussion | Reported. |
| 20 (D;V) | Discuss the potential clinical use of the model and implications for future research. | 4 Discussion; 5 Conclusion | Reported. |
| 21 (D;V) | Provide information about the availability of supplementary resources, such as study protocol, web calculator, and datasets. | Data and code availability | Reported. |
| 22 (D;V) | Provide the source of funding and the role of the funders for the study. | Funding | Reported. |

Note: TRIPOD, Transparent Reporting of a multivariable prediction model for Individual Prognosis Or Diagnosis; D, development; V, validation; CI, confidence interval; CRRT, continuous renal replacement therapy; KRT, kidney replacement therapy. “Reported” indicates that the item was fully reported, “partially reported” indicates partial reporting, and “not applicable” indicates that the item did not apply to this study.

Supplementary Table 6. PROBAST risk-of-bias and applicability assessment for model development and temporal validation

| Evaluation | Domain/item | Official signaling question | Judgment | Rationale and domain/overall judgment |
| --- | --- | --- | --- | --- |
| Model development | Participants 1.1 | Were appropriate data sources used, for example cohort, randomized controlled trial, or nested case-control study data? | Y | Retrospective cohort data were appropriate for model development. |
| Model development | Participants 1.2 | Were all inclusions and exclusions of participants appropriate? | PY | Eligibility criteria were explicit, but restricting the study to planned discontinuation attempts may have selected a clinically narrower population. Domain judgment: high risk of bias; high concern for applicability. |
| Model development | Predictors 2.1 | Were predictors defined and assessed in a similar way for all participants? | Y | Definitions, t0, units, and measurement windows were standardized. |
| Model development | Predictors 2.2 | Were predictor assessments made without knowledge of outcome data? | Y | Predictors were recorded before the future 7-day outcome; adjudicators did not use model probabilities. |
| Model development | Predictors 2.3 | Are all predictors available at the time the model is intended to be used? | Y | All six predictors were available before CRRT discontinuation. Domain judgment: low risk of bias; low concern for applicability. |
| Model development | Outcome 3.1 | Was the outcome determined appropriately? | Y | Survival for 7 days without any KRT was clinically relevant and fully ascertainable. |
| Model development | Outcome 3.2 | Was a prespecified or standard outcome definition used? | Y | The same prespecified 7-day event definition was applied. |
| Model development | Outcome 3.3 | Were predictors excluded from the outcome definition? | Y | Urine output and creatinine were removed from the outcome definition. |
| Model development | Outcome 3.4 | Was the outcome defined and determined in a similar way for all participants? | Y | KRT records and death records were assessed consistently. |
| Model development | Outcome 3.5 | Was outcome determination made without knowledge of predictor information? | PN | The clinical team knew physiological information when deciding KRT restart, leading to decision-dependent outcome determination. |
| Model development | Outcome 3.6 | Was the time interval between predictor assessment and outcome determination appropriate? | Y | Predictors were measured before t0, and outcome assessment continued for 7 days. Domain judgment: high risk of bias; low concern for applicability. |
| Model development | Analysis 4.1 | Were there a reasonable number of participants with the outcome? | Y | The development cohort included 139 failures for six parameters. |
| Model development | Analysis 4.2 | Were continuous and categorical predictors handled appropriately? | Y | Functional form was evaluated for continuous variables, and categorical predictors were prespecified. |
| Model development | Analysis 4.3 | Were all enrolled participants included in the analysis? | Y | All eligible first attempts were analyzed after multiple imputation; repeated attempts were assessed separately. |
| Model development | Analysis 4.4 | Were participants with missing data handled appropriately? | Y | Multiple imputation and complete-case/deployment-rule sensitivity analyses were reported. |
| Model development | Analysis 4.5 | Was selection of predictors based on univariable analysis avoided? | Y | Predictor selection based on univariable analysis was avoided. |
| Model development | Analysis 4.6 | Were complexities in the data, such as censoring, competing risks, or case-control sampling, accounted for appropriately? | PY | Composite events and repeated attempts were addressed in sensitivity analyses, although residual complexity remained. |
| Model development | Analysis 4.7 | Were relevant model performance measures evaluated appropriately? | Y | Discrimination, calibration, overall accuracy, classification performance, and net benefit were evaluated. |
| Model development | Analysis 4.8 | Were model overfitting and optimism in model performance accounted for? | Y | Bootstrap optimism correction and uniform shrinkage were used. |
| Model development | Analysis 4.9 | Do predictors and their assigned weights in the final model correspond to the results from multivariable analysis? | Y | Final coefficients corresponded to the multivariable model after prespecified shrinkage. Domain judgment: low risk of bias. Overall risk of bias: high; overall concern for applicability: high. |
| Temporal validation | Participants 1.1 | Were appropriate data sources used, for example cohort, randomized controlled trial, or nested case-control study data? | Y | A later calendar-period cohort was appropriate for temporal validation. |
| Temporal validation | Participants 1.2 | Were all inclusions and exclusions of participants appropriate? | PY | Eligibility criteria were unchanged, but planned discontinuation selection at a single center limited transportability. Domain judgment: high risk of bias; high concern for applicability. |
| Temporal validation | Predictors 2.1 | Were predictors defined and assessed in a similar way for all participants? | Y | The same definitions, t0, and measurement windows were used. |
| Temporal validation | Predictors 2.2 | Were predictor assessments made without knowledge of outcome data? | Y | Predictors were recorded before the future 7-day outcome. |
| Temporal validation | Predictors 2.3 | Are all predictors available at the time the model is intended to be used? | Y | All predictors were available before the intended time of use. Domain judgment: low risk of bias; low concern for applicability. |
| Temporal validation | Outcome 3.1 | Was the outcome determined appropriately? | Y | The same clinically relevant 7-day outcome was used. |
| Temporal validation | Outcome 3.2 | Was a prespecified or standard outcome definition used? | Y | The development-stage definition was retained without change. |
| Temporal validation | Outcome 3.3 | Were predictors excluded from the outcome definition? | Y | The outcome did not include predictor components. |
| Temporal validation | Outcome 3.4 | Was the outcome defined and determined in a similar way for all participants? | Y | Outcome determination was the same as in the development stage. |
| Temporal validation | Outcome 3.5 | Was outcome determination made without knowledge of predictor information? | PN | KRT restart remained a clinician-mediated decision influenced by physiological variables. |
| Temporal validation | Outcome 3.6 | Was the time interval between predictor assessment and outcome determination appropriate? | Y | Predictor assessment preceded the 7-day outcome. Domain judgment: high risk of bias; low concern for applicability. |
| Temporal validation | Analysis 4.1 | Were there a reasonable number of participants with the outcome? | Y | The validation cohort included 67 failures, and uncertainty was reported. |
| Temporal validation | Analysis 4.2 | Were continuous and categorical predictors handled appropriately? | Y | Locked coding and transformations were retained. |
| Temporal validation | Analysis 4.3 | Were all enrolled participants included in the analysis? | Y | All 143 validation participants were included after multiple imputation. |
| Temporal validation | Analysis 4.4 | Were participants with missing data handled appropriately? | Y | Validation-cohort multiple imputation and alternative missing-data analyses were reported. |
| Temporal validation | Analysis 4.5 | Was selection of predictors based on univariable analysis avoided? | NA | No predictor selection was performed in the validation data. |
| Temporal validation | Analysis 4.6 | Were complexities in the data, such as censoring, competing risks, or case-control sampling, accounted for appropriately? | PY | Temporal drift, protocol adherence, and subgroups were examined, but sample size was limited. |
| Temporal validation | Analysis 4.7 | Were relevant model performance measures evaluated appropriately? | Y | AUC, calibration, Brier score, classification measures, and DCA were evaluated. |
| Temporal validation | Analysis 4.8 | Were model overfitting and optimism in model performance accounted for? | NA | No model development or coefficient estimation was performed in the validation cohort; uncertainty was estimated with patient-level bootstrap. |
| Temporal validation | Analysis 4.9 | Do predictors and their assigned weights in the final model correspond to the results from multivariable analysis? | NA | The final development equation and threshold were applied directly without updating. Domain judgment: low risk of bias. Overall risk of bias: high; overall concern for applicability: high. |

Note: Y, yes; PY, probably yes; PN, probably no; N, no; NI, no information; NA, not applicable; PROBAST, Prediction model Risk Of Bias ASsessment Tool; RCT, randomized controlled trial; AUC, area under the receiver operating characteristic curve; DCA, decision curve analysis; CRRT, continuous renal replacement therapy; KRT, kidney replacement therapy. t0 denotes the actual CRRT discontinuation time. Official signaling questions are presented in English for readability; model development and temporal validation were assessed separately, with domain-level and overall judgments presented in the same table.
